# Strategies to Enhance the Efficacy and Clinical Translation of Antimicrobial Photodynamic Therapy

**DOI:** 10.3390/antibiotics15080748

**Published:** 2026-08-02

**Authors:** Zixing Lin, Qianhui You, Haohui Zhu, Ziya Lao, Jiaying Lao, Xiting Li, Xuechao Yang, Min Nie

**Affiliations:** 1 Guangdong Engineering Research Center of Oral Restoration and Reconstruction, Guangzhou Key Laboratory of Basic and Applied Research of Oral Regenerative Medicine, Department of Basic Oral Medicine, School of Stomatology, Guangzhou Medical University, Guangzhou 510182, China; 2022119085@stu.gzhmu.edu.cn (Z.L.); 2025211625@stu.gzhmu.edu.cn (Q.Y.); haohuizhu@stu.gzhmu.edu.cn (H.Z.); 2023119058@stu.gzhmu.edu.cn (Z.L.); jiaying_lao@163.com (J.L.); 2 Hospital of Stomatology, Sun Yat-sen University, Guangzhou 510055, China; 3 Guangdong Engineering Research Center of Oral Restoration and Reconstruction, Guangzhou Key Laboratory of Basic and Applied Research of Oral Regenerative Medicine, Department of General Dentistry Center, School of Stomatology, Guangzhou Medical University, Guangzhou 510182, China

**Keywords:** photosensitizer, nanomaterial, ROS, light source

## Abstract

**Background:** Antimicrobial resistance represents a growing global health challenge, necessitating the development of effective non-antibiotic antimicrobial approaches. Antimicrobial photodynamic therapy (aPDT) has emerged as a promising localized antimicrobial strategy owing to its broad-spectrum activity, biofilm-targeting capability, and low propensity to induce resistance. However, its clinical translation remains restricted by limited photosensitizer (PS) performance, insufficient light penetration, oxygen dependency, biofilm-associated barriers, and the lack of standardized treatment protocols. **Methods:** This narrative review summarizes recent strategies developed to enhance the efficacy and translational potential of aPDT, including PS engineering, nanomaterial- and non-nanomaterial-based delivery systems, advanced light-source technologies, hypoxia-modulating approaches, and synergistic therapeutic strategies. In addition, current challenges associated with regulatory approval, manufacturing scalability, treatment standardization, and clinical implementation are discussed. **Results:** Recent advances have transformed aPDT from a conventional PS–light–oxygen system into a multifunctional antimicrobial platform. Emerging approaches improve bacterial targeting, biofilm penetration, reactive oxygen species generation, oxygen utilization, and therapeutic precision. Nevertheless, many advanced systems remain at the preclinical stage due to complexity, cost, safety concerns, and insufficient clinical validation. **Conclusions:** aPDT should be considered a targeted therapeutic option for accessible, localized, and biofilm-associated infections rather than a replacement for systemic antimicrobial therapy. Future clinical translation will depend on balancing technological innovation with biosafety, scalability, and protocol standardization. Strategies integrating intelligent PS design, oxygen regulation, and clinically feasible synergistic approaches may provide promising pathways toward the broader application of aPDT in antimicrobial management.

## 1. Background

Bacterial infectious diseases remain a major threat to global public health and are predominantly managed with antibiotic therapy [1]. However, the widespread and often inappropriate use of antibiotics has accelerated the emergence of antimicrobial resistance, which is now recognized as one of the most pressing challenges in modern medicine [2,3]. Consequently, there is an urgent need to develop alternative antimicrobial strategies that are less prone to inducing resistance. Antimicrobial photodynamic therapy (aPDT) has emerged as a promising non-antibiotic approach because of its broad-spectrum antimicrobial activity, favorable safety profile, and low likelihood of inducing bacterial resistance [4]. It is worth noting that photodynamic therapy’s most established and clinically mature application to date has been in oncology, where it is an approved treatment for several neoplastic and pre-neoplastic conditions. As an extension to microbial infections, aPDT represents a comparatively narrower and more recently developed application, one whose realistic clinical scope is further shaped by the fact that most microbial infections, unlike many localized tumors, are systemic or deep-seated in nature. Nevertheless, despite decades of research and encouraging preclinical findings, the clinical application of aPDT remains limited. Recent clinical practice guidelines have questioned the routine use of adjunctive photodynamic therapy in certain clinical settings due to insufficient evidence of additional clinical benefit and concerns regarding cost-effectiveness [5,6]. These limitations highlight the need for further technological improvements to enhance the efficacy and translational potential of aPDT.

Despite its attractive antimicrobial properties, the clinical application of aPDT is still limited by poor bacterial selectivity, photosensitizer aggregation, insufficient tissue penetration, hypoxia, and biofilm-associated resistance [7]. Consequently, recent research has increasingly focused on developing strategies that address these barriers instead of merely demonstrating the antibacterial efficacy of aPDT [4].

While systemic antibiotics remain more effective for invasive or deep-seated infections, their efficacy against biofilm-embedded or multidrug-resistant organisms in accessible sites is often limited, a gap that aPDT is specifically positioned to address. Given these considerations, aPDT is best conceptualized not as a replacement for systemic antibiotic therapy but as a targeted option for specific clinical scenarios: superficial or readily light-accessible infections, such as chronic wounds and dental/periodontal biofilms [5,6]; infections caused by multidrug-resistant organisms for which antibiotic options are increasingly limited [3,4]; and biofilm-associated infections, where the protective extracellular matrix substantially reduces antibiotic penetration and efficacy [8]. In such settings, aPDT’s low propensity to induce further resistance [4] offers a meaningful advantage over repeated antibiotic courses. Conversely, for systemic bacteremia or deep-seated infections, established antimicrobial therapy remains the standard of care, and aPDT is not currently intended as a substitute.

**Figure 1:**
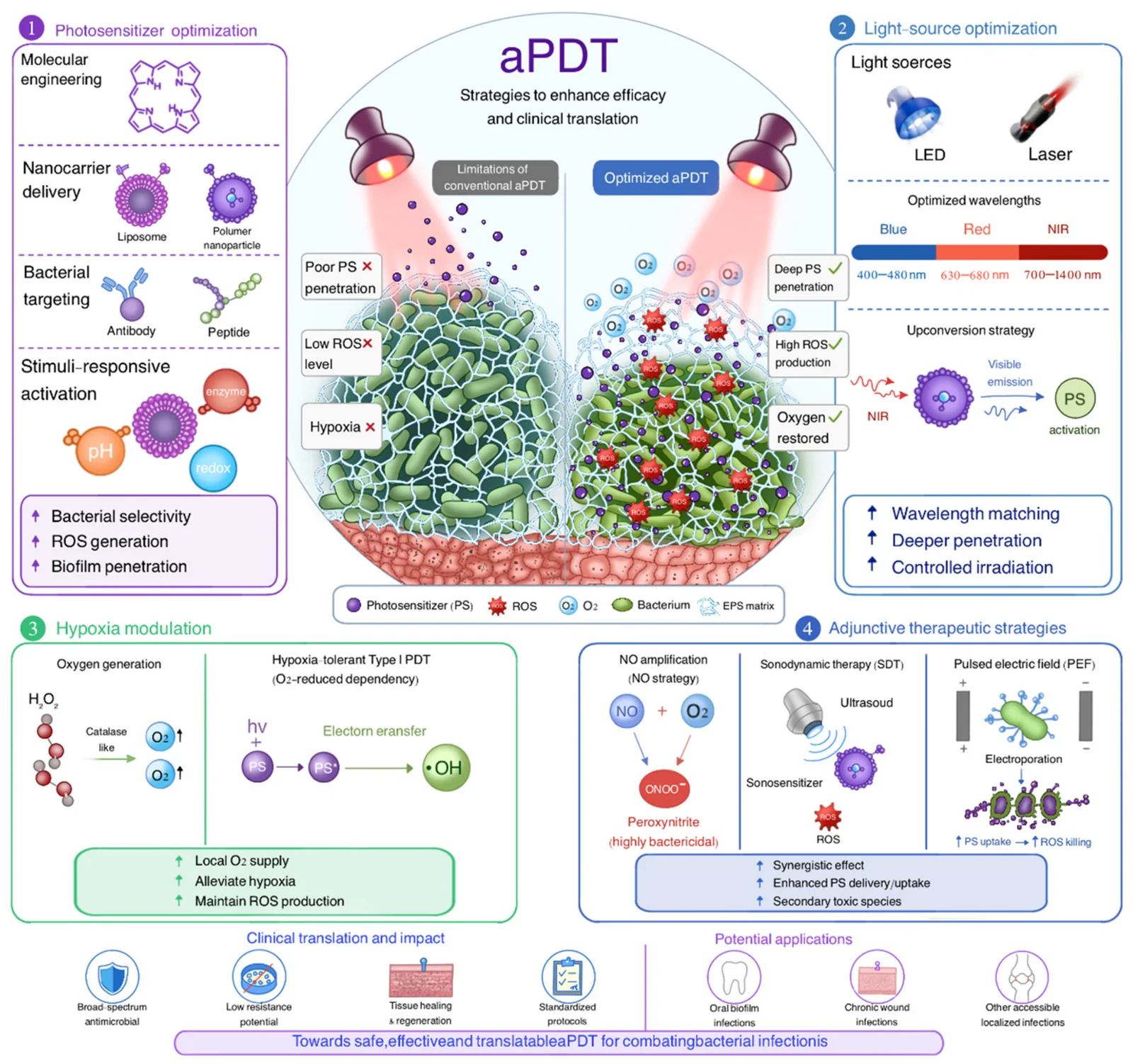
Overall framework of strategies for enhancing antimicrobial photodynamic therapy (aPDT). Conventional aPDT is limited by insufficient photosensitizer penetration, hypoxia, limited light penetration, and inadequate ROS generation. The four major optimization strategies discussed in this review, including photosensitizer optimization, light-source optimization, hypoxia modulation, and adjunctive therapeutic strategies, collectively improve antibacterial efficacy and promote the clinical translation of aPDT. Purple arrows indicate light irradiation, and asterisks (*) denote excited photosensitizers (PS*).

Accordingly, this narrative review summarizes recent strategies aimed at overcoming the major barriers to aPDT efficacy and clinical translation. As illustrated in Figure 1, these strategies are organized into four principal categories: (i) PS optimization, (ii) light-source optimization, (iii) hypoxia modulation, and (iv) adjunctive therapeutic strategies. In addition, this review discusses the remaining barriers to clinical implementation and highlights future directions for the translation of next-generation aPDT.

## 2. PS Optimization

PSs are the core component of aPDT because they determine ROS generation, bacterial selectivity, and ultimately the antimicrobial efficacy of aPDT. Consequently, optimizing PS performance has become one of the major strategies for enhancing the efficacy and clinical translation of aPDT [9].

Although successive generations of PSs have substantially improved photodynamic performance, currently available PSs still suffer from several intrinsic limitations, including insufficient bacterial targeting, poor aqueous solubility, self-aggregation, limited biofilm penetration, and suboptimal ROS generation [10]. Therefore, extensive efforts have focused on optimizing PSs through structural modification, nanomaterial-assisted delivery, and non-nanomaterial engineering strategies, which are discussed in the following sections [7].

### 2.1. Structural Optimization of PS

PSs are the core component of aPDT because they determine ROS generation, bacterial selectivity, and ultimately the antimicrobial efficacy of aPDT [11]. Consequently, optimizing PS performance has become one of the major strategies for enhancing the efficacy and clinical translation of aPDT [12,13]. Although successive generations of PSs have substantially improved photodynamic performance, currently available PSs still suffer from several intrinsic limitations, including insufficient bacterial targeting, poor aqueous solubility, self-aggregation, limited biofilm penetration, and suboptimal ROS generation [11].

The evolution of PSs has been driven primarily by the need to improve ROS generation, bacterial selectivity, and biocompatibility while reducing off-target toxicity. Accordingly, PSs have progressed through three generations with progressively improved photophysical and biological properties. First-generation PSs, represented by hematoporphyrin derivatives, demonstrated the feasibility of aPDT but were limited by low chemical purity, prolonged skin photosensitivity, and relatively poor ROS generation. Second-generation PSs, including porphyrins, phthalocyanines, chlorins, phenothiazinium dyes, and related compounds, improved absorption characteristics, ROS production, and tissue selectivity. More recently, third-generation PSs have incorporated targeting ligands, antibodies, peptides, glycans, or functional carriers to further enhance bacterial specificity and reduce off-target effects. Nevertheless, most third-generation PSs remain at the preclinical stage due to manufacturing complexity, high production costs, and limited clinical evidence [9,11].

An ideal PS for aPDT should possess high bacterial selectivity, favorable physicochemical properties, and excellent photodynamic performance. Specifically, it should exhibit appropriate hydrophilicity, positive charge, and small molecular size to facilitate bacterial binding and biofilm penetration, while maintaining a high triplet quantum yield for efficient ROS generation. In addition, an ideal PS should demonstrate high photostability, low dark toxicity, good solubility, and minimal self-aggregation to maximize the therapeutic window. Rapid clearance after treatment is also desirable to reduce prolonged photosensitivity and improve clinical safety [14].

Based on their chemical structure and origin [13,14], PSs used in aPDT can be broadly classified into tetrapyrrole compounds (porphyrin [15,16], chlorin [17,18], phthalocyanine [19], etc.), synthetic dyes (phenothiazinium [13], squaraine [20], BODIPY dyes [21], phenalenone [22], etc.), natural PSs (riboflavin [23], curcumin [24,25], anthraquinones [26,27,28], hypericin [29], etc.), and nanostructured PS or nanoformulations [13].

Different PS categories exhibit distinct absorption spectra, ROS-generation efficiency, and bacterial affinity. Currently, aPDT has been mainly investigated for the treatment of oral bacterial diseases, particularly biofilm-associated infections. The commonly used PSs in clinical treatment are toluidine blue (TB) [30,31], methylene blue (MB) [30,32], curcumin [24,25], indocyanine green (ICG) [33], riboflavin [25], rose Bengal [34,35]. Among them, MB is the most commonly used PS, while ICG is considered very promising due to its good tissue penetration secondary to a laser with a wavelength of 810 nm [33]. Many PSs are still in the primary and animal study phases. The rational design and optimization of PSs with enhanced ROS generation, bacterial targeting ability, and biocompatibility represent important directions for developing more effective aPDT.

Despite these advances, the clinical translation of second- and third-generation PSs remains challenged by both the intrinsic limitations of aPDT as a localized therapy and the practical constraints associated with advanced PS development. Third-generation PSs conjugated with antibodies or targeting peptides substantially increase synthesis complexity and production cost, and their large-scale manufacturing under good manufacturing practice standards remains largely unexplored. Moreover, most comparative data on PS performance derive from in vitro planktonic assays; how these properties translate to complex, protein-rich biological fluids and mixed-species biofilms in vivo remains poorly characterized.

### 2.2. PS and Nanomaterial

Nanomaterials contribute to aPDT through two distinct roles [36,37]; they can either function as intrinsic photosensitizers that directly generate ROS or serve as nanocarriers to improve the delivery, stability, and targeting ability of conventional photosensitizers. The following nanomaterial platforms are commonly used in aPDT:

- Liposomes
- Polymer-based nanoparticles: chitosan, cationic polylactic acid-acetic acid copolymer, nanospheres, nanocapsules, micelles
- Nanodiamonds
- Nanoemulsion
- Silica-based nanoparticles
- Metal oxide nanoparticles (zinc oxide nanoparticles, gold nanoparticles, silver nanoparticles, titanium dioxide, magnetic nanoparticles (MNPs, Fe_3_O_4_), etc.)
- Upconversion nanoparticles (UCNPs) (solid-state materials doped with lanthanide ions, etc.)
- Carbon nanoparticles (graphene quantum dots/graphene and carbon nanotubes)
- Fullerene

Some nanomaterials mentioned above can also be used simultaneously as PS or PS carriers. Nanoparticles improve ROS production and bioavailability and avoid host toxicity in aPDT through the following two aspects [13].

#### 2.2.1. Nanomaterials Improve PS Targeting

**Figure 2:**
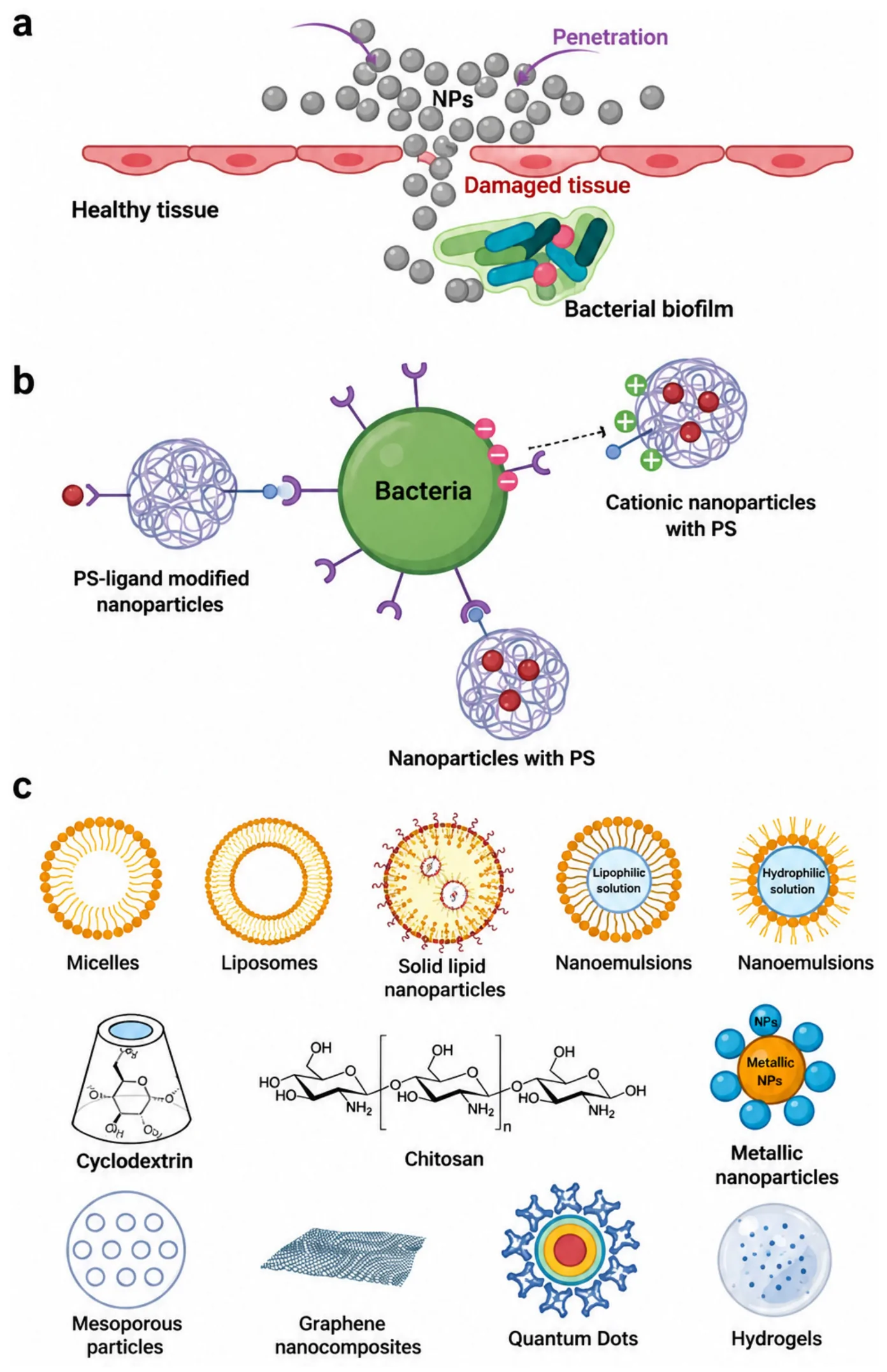
Schematic illustration of nanoplatform-based aPDT: (**a**) passive targeting of PS, (**b**) active targeting of PS, and (**c**) nanomaterial for PS loading.

Nanoplatform-based targeting can be divided into passive (Figure 2a) and active (Figure 2b). Passive targeting is based on the principle that blood vessels of injured tissue are more permeable than normal vessels, so nanoparticles with appropriate size, shape, material, and surface modification will deposit more easily in injured tissue [38]. The disadvantages of passive targeting are failure to selectively target bacteria, toxicity to host tissue, and loss of blood vessel permeability in the later stage of infection. Therefore, passive targeting only appears in the early stages of acute infection [38]. The principle of active targeting is to modify nanomaterials with ligands or mediators that selectively bind bacteria, then combine them with PS to target specific bacteria. Recognizing substances between modified PS and bacteria can be divided into low-selective charge and high-selective antibodies, peptides, glycans, etc [38]. Nanomaterials used for aPDT can be broadly divided into the following five categories [39]:

- Micelles and liposomes: Their structures allow encapsulation of lipophilic drugs in the core of micelles and the membrane of liposomes or hydrophilic molecules in the core of liposomes. Tailoring the surface properties of micelles and lipids can increase the specificity of target bacteria.
- Bio-sourced oligosaccharide conjugates: The surface of nanoparticles can be modified with glycans to mimic the lectin on the surface of host cells, recognizing bacteria, thereby targeting and recognizing bacteria.
- Inorganic nanoparticles: Core–shell hybrid structures can be arranged, and different material coatings (e.g., UCNPs) can enhance PS targeting by leveraging their unique electronic, physical, and morphological properties.
- Antibody-modified nanoparticles with PS can recognize bacteria with high selectivity via antigen–antibody interactions.
- Synthetic and other bio-inspired polymer conjugates: Hyperbranched macromolecules and dendrimers target bacteria by adding PSs and introducing a particular conjugate formed by the outer cation layer. In addition, reticular polymer networks form “nanodots” with inherent luminescence properties and improve bacterial targeting by further modifying the surface.

Overall, nanomaterial-assisted targeting strategies enhance the antibacterial efficacy of aPDT by increasing PS accumulation at infection sites through either passive localization or active bacterial recognition. Compared with passive targeting, active targeting generally offers greater bacterial specificity and therefore holds greater promise for clinical translation.

#### 2.2.2. Nanomaterials Address the Hydrophobicity and Self-Aggregation of PS

Most PSs are hydrophobic and self-aggregating, which facilitates their easy clearance by the immune system in vivo and reduces their bioavailability [40]. Therefore, many nanomaterials, including liposomes and micelles, are used to construct drug delivery systems that combine the modification or encapsulation of PSs with improving their bioavailability. Lipids tend to aggregate and form bilayers in an aqueous environment spontaneously. The hydrophilic PS is suspended in an aqueous environment along with other compounds, then placed in the center of the liposome. The hydrophobic PS dissolves in a hydrophobic environment, producing liposomes containing PS within the lipid bilayer. Smaller than liposomes and can be treated more effectively, micelles are aggregates of surfactants or block polymers that self-assemble in an aqueous solution and spontaneously form nanostructures (particle size 5–100 nm) under certain conditions. In addition, all of these have the advantage of being cheaper and easier to prepare [41].

In addition to liposomes and micelles, many other nanomaterials are also used to modify PS to address their hydrophobicity and self-aggregation, such as solid lipid nanoparticles, emulsions, cyclodextrins, chitosan, metal nanoparticles, porous materials (mainly carbon, silicon dioxide, metal oxides), graphene nanocomposites, quantum dots, films, hydrogels, and other nanomaterials [40] (Figure 2c).

Collectively, these nanocarrier systems primarily improve aPDT by enhancing PS solubility, preventing self-aggregation, and increasing bioavailability, thereby facilitating more efficient ROS generation and antibacterial activity.

#### 2.2.3. Stimuli-Responsive Nanomaterials

Stimuli-responsive nanomaterials have recently attracted increasing attention as advanced delivery systems for aPDT [42]. Unlike conventional nanocarriers, these smart platforms can respond to endogenous stimuli within the infectious microenvironment, such as acidic pH, redox imbalance, and enzyme overexpression, or to exogenous triggers including light, heat, and ultrasound, thereby enabling site-specific activation and controlled release of PSs [43]. Such strategies improve PS accumulation, biofilm penetration, and ROS generation while minimizing off-target effects [44].

To illustrate this concept, recent studies have reported the development of specific stimuli-responsive platforms tailored to the pathological characteristics of bacterial infections.

- pH-Responsive Systems: The acidic microenvironment of bacterial biofilms (typically pH 4.5–6.5) can be exploited to trigger drug release or enhance targeting. For example, researchers have designed charge-reversal polymeric nanocarriers that switch their surface charge from negative to positive upon entering the acidic biofilm microenvironment. This pH-triggered charge reversal facilitates strong electrostatic interactions with the negatively charged bacterial cell walls. As a result, the nanoplatforms achieve deeper biofilm penetration and selective PS accumulation, leading to the eradication of over 99% of *Staphylococcus aureus* biofilms in vitro and significantly accelerating healing in in vivo infected wound models [45].
- Enzyme-Responsive Systems: Bacterial biofilms often overexpress specific enzymes, such as hyaluronidase or lipase, to maintain their extracellular polymeric substance. A classic example involves hyaluronic acid-modified nanoparticles loaded with a PS. When these nanoparticles encounter the biofilm, bacteria-secreted hyaluronidase specifically cleaves the hyaluronic acid coating. This enzymatic degradation not only triggers the on-demand release of PS but also physically disrupts the EPS matrix’s structural integrity. Experimental results demonstrate that this dual-action mechanism allows the PS to penetrate deeply into mature *Pseudomonas aeruginosa* biofilms, reducing bacterial viability by several logs more than non-responsive PS controls [46].
- Redox-Responsive Systems: Bacteria often maintain elevated intracellular glutathione (GSH) levels to defend against oxidative stress. To counter this, disulfide-bond-bridged nanoplatforms have been synthesized. Upon cellular uptake, the high intracellular GSH concentration cleaves the disulfide bonds, causing the nanostructure to disassemble and release the PS. More importantly, this redox reaction consumes the bacteria’s endogenous GSH. The depletion of the bacteria’s primary antioxidant defense mechanism drastically amplifies their vulnerability to aPDT-generated ROS. Studies have shown that this GSH-depleting strategy significantly enhances the bactericidal efficacy against multidrug-resistant strains while exhibiting minimal toxicity to normal mammalian cells [46].

Beyond single-stimulus responsiveness, multifunctional nanoplatforms capable of responding to exogenous triggers such as near-infrared (NIR) irradiation, heat, or ultrasound have been developed to integrate aPDT with photothermal therapy, chemodynamic therapy, sonodynamic therapy, and biofilm-disrupting strategies [47]. For instance, NIR-responsive nanocarriers not only achieve deep tissue penetration and spatiotemporally controlled PS activation but also generate localized hyperthermia that disrupts EPS integrity and enhances bacterial membrane permeability, thereby synergistically amplifying ROS-mediated bacterial killing [42]. Concurrently, ultrasound-triggered sonosensitizers can penetrate deeply into biofilms where light cannot reach, producing ROS via acoustic cavitation and mechanically disrupting the dense EPS matrix. Furthermore, stimuli-responsive systems have been engineered to co-deliver PSs with EPS-degrading enzymes or quorum-sensing inhibitors, achieving concurrent biofilm dispersion and bacterial eradication. These multi-modal platforms collectively address the limitations of standalone aPDT by overcoming biofilm penetration barriers, compensating for oxygen dependence, and broadening the antimicrobial spectrum, positioning them as next-generation intelligent systems for combating recalcitrant biofilm-associated infections [48].

Collectively, these examples demonstrate that activatable PS capable of responding to bacterial microenvironmental cues have become an important direction for improving the precision and therapeutic efficacy of aPDT [48].

### 2.3. PS with Non-Nanomaterials

In addition to nanomaterials, a variety of non-nanomaterial approaches have been developed to improve the performance of PS. Unlike nanocarrier-based systems, these strategies primarily act by (i) enhancing bacterial selectivity, (ii) increasing intracellular PS accumulation, or (iii) improving PS delivery and stability. Accordingly, they can be grouped according to their primary mechanism of action rather than their material type [39].

#### 2.3.1. Strategies for Improving Bacterial Selectivity

- Small cationic groups: Small cationic groups, such as amino-functionalized groups, are conjugated for electrostatic interactions and coupled to PS that target negatively charged bacterial membranes.
- Antibiotics: Antibiotics and PS conjugation exhibit high target selectivity and killing efficacy by targeting and disrupting the membrane integrity of microorganisms. They do not involve resistance mechanisms because the antibiotic concentration is deficient and is not directly responsible for sterilization.
- Antimicrobial peptide conjugates: This linear or cyclic amphipathic peptide can be bactericidal by targeting bacterial membranes electrostatically, inserting into and disrupting cell membranes, or interfering with cellular metabolic function.
- Metal complexes: Metal complexes exhibit excellent photovoltaic properties and can enhance aPDT activity through modulation of ligands, metal centers, and bacterial targeting to achieve selective toxicity efficacy [49].

Collectively, these approaches primarily enhance bacterial selectivity, thereby increasing PS accumulation at the infection site while minimizing damage to surrounding host tissues.

#### 2.3.2. Strategies for Increasing Intracellular PS Accumulation

Unlike targeting strategies, Efflux pump inhibitors (EPI) combined with PS can suppress outward transport processes and increase the concentration of intracellular PSs, thereby enhancing phototoxicity in bacterial cells, reducing the damaging effects on neighboring host cells exposed to deleterious ROS, and improving the efficacy of aPDT [50].

#### 2.3.3. Strategies for Improving PS Delivery

Exosomes, which inherently contain bactericidal proteins and antimicrobial peptides, not only play an essential role in intercellular communication but also serve as biologically derived delivery platforms that improve the transport and biocompatibility of hydrophobic PS via direct mixing, thereby enhancing the efficiency of aPDT [51]. However, their application in antimicrobial PDT remains at an early stage.

Taken together, non-nanomaterial strategies offer relatively simple, versatile approaches to enhance aPDT efficacy. Among them, bacterial-targeting conjugates appear to have the greatest translational potential because they improve antimicrobial selectivity without substantially increasing formulation complexity, whereas exosome-based delivery systems remain promising but require further optimization for large-scale production and clinical application.

Non-nanomaterial strategies are not free of caveats. Antibiotic–PS conjugates, although designed to act via non-resistance-inducing mechanisms, still introduce sub-lethal antibiotic exposure into the microenvironment, and the long-term risk of selecting for antibiotic cross-resistance has not been rigorously excluded. Efflux pump inhibitors may themselves exhibit host–cell toxicity at effective concentrations, and exosome-based delivery, while biocompatible, currently lacks standardized, scalable isolation and purification protocols suitable for clinical-grade production.

## 3. Light-Source Optimization

Activating PS requires a light source, and the ideal light source should meet the following conditions:

- The light wavelength matches the absorption peak of PS
- Adequate power and irradiation
- The intensity of the spot is evenly distributed
- Efficient light transmission mode (through optical fiber or direct irradiation) [52]

The following improvements were studied from the light source’s wavelength and carrier.

### 3.1. Improvement of Light Source Wavelength

The light sources commonly used in aPDT are primarily located in the blue (400–480 nm) and red (630–680 nm) regions of the visible spectrum [14]. Within the visible and near-infrared ranges relevant to aPDT, longer wavelengths generally provide greater tissue penetration. Blue light has been reported to exhibit intrinsic antimicrobial effects under certain conditions, mainly through excitation of endogenous bacterial chromophores and subsequent generation of reactive oxygen species [53]. However, its limited tissue penetration restricts its application to superficial infections. In contrast, red light provides deeper tissue penetration but generally lacks direct antibacterial activity in the absence of exogenous photosensitizers because of its lower photon energy and limited interaction with endogenous bacterial components. Therefore, photosensitizer-mediated conversion of light energy into cytotoxic reactive species remains essential for most red-light-based aPDT applications. To address these limitations, investigators have developed the following strategies.

#### 3.1.1. Upconversion Luminescence-Based aPDT

Although several long-wavelength/NIR-absorbing PS (e.g., phthalocyanines and cyanine derivatives) can directly utilize deeply penetrating light, their clinical translation is frequently hindered by severe hydrophobic aggregation, rapid photobleaching, and compromised singlet oxygen ($\Phi_{\Delta }$) yields in physiological aqueous environments. To circumvent the reliance on these aggregation-prone NIR PSs, UCNPs offer an indirect activation strategy by converting deeply penetrating NIR light into higher-energy visible or ultraviolet emission in situ [54]. This energy transfer enables the secondary activation of classical, highly potent PS (such as TiO_2_ or Rose Bengal) that possess high ROS quantum yields but otherwise require shallow-penetrating UV/visible excitation, thereby effectively coupling deep tissue penetration with enhanced photochemical efficacy [55].

**Figure 3:**
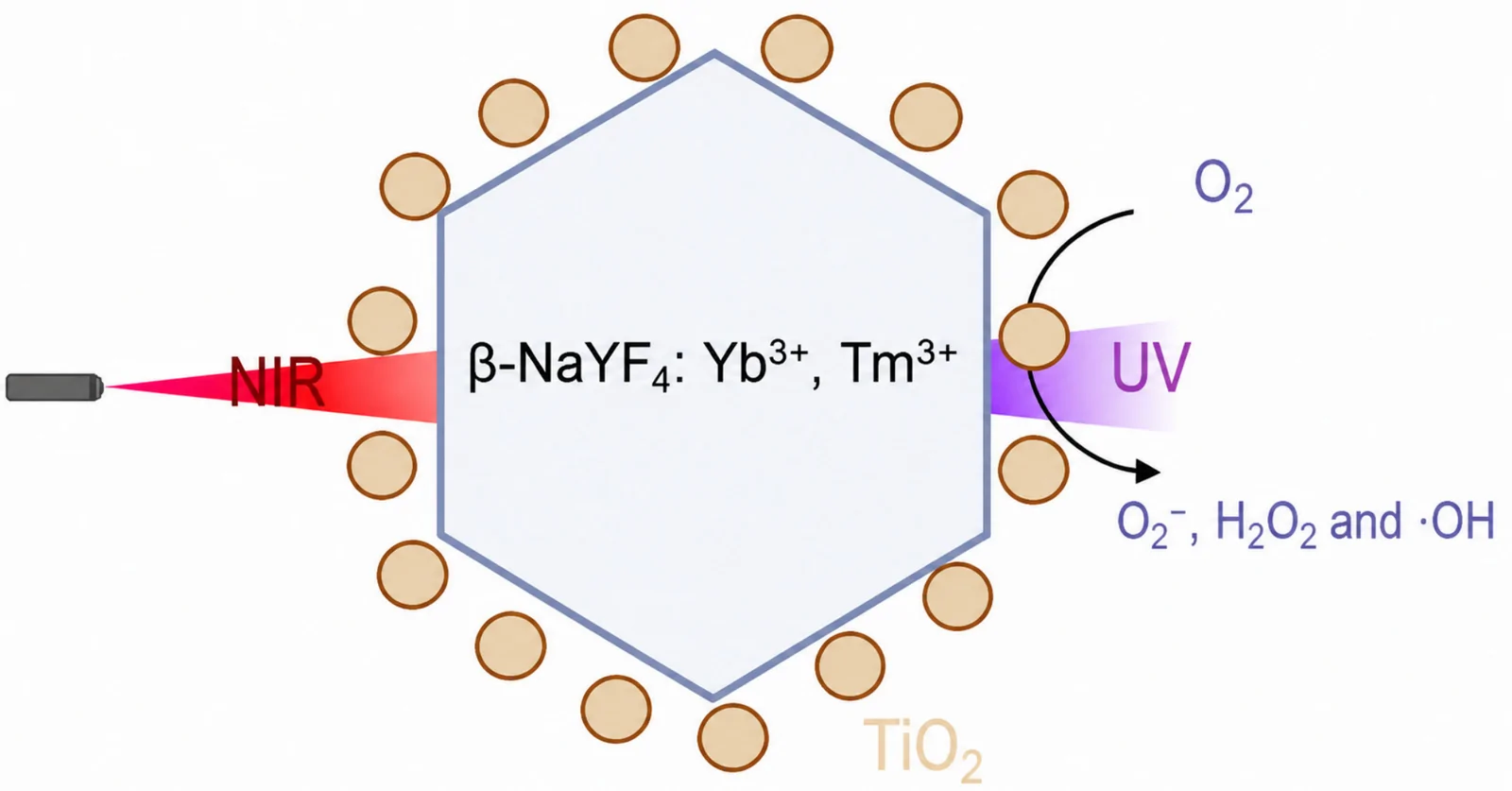
The core–shell structure of UCNPs@TiO_2_ converts incident NIR light into UV light.

A representative implementation was demonstrated by Qi et al. [56], who first synthesized UCNPs@TiO_2_ with a core–shell structure, in which β-NaYF_4_: Yb^3+^, Tm^3+^ composes the core, TiO_2_ is the shell, and this hexagonal structure converts incoming NIR into UV, triggers TiO_2_ to produce ROS to kill bacteria in the deep layers of the tissue, and shows a solid bactericidal ability against periodontitis-related pathogens in the planktonic and biofilm stages (Figure 3).

#### 3.1.2. Replacement of Specific Wavelengths with White Light

Daylight aPDT (DL-aPDT) is a new aPDT that has emerged recently. It uses daylight as its light source and is low-cost, so it can be done at home or in a clinic. DL-aPDT is often used to treat actinic keratosis [57]. Although aPDT commonly uses wavelength-specific light sources to maximize photosensitizer activation, broad-spectrum illumination has also been explored, particularly in daylight-mediated PDT. In these approaches, sunlight or artificial broad-spectrum light sources, such as metal halide lamps, are used to activate photosensitizers with relatively broad absorption bands. However, the clinical application of sunlight-based PDT remains limited because of variations in irradiance, weather conditions, and difficulties in controlling the delivered light dose. Perez-Laguna et al. compared red LED irradiation (625 nm) with broad-spectrum white light irradiation (420–700 nm) generated by a metal halide lamp for methylene blue-mediated inactivation of *Candida albicans* (*C. albicans*) [58]. Both light sources achieved comparable antifungal efficacy; notably, the broad-spectrum lamp reduced the required irradiation time, likely due to its higher irradiance and ability to deliver the required fluence more rapidly.

### 3.2. The Light Source for aPDT

The type of light source, its power, irradiation time, energy, spot size, distance from the target, and the applied technique are all important factors affecting the efficacy of aPDT [33]. There are three primary sources of light for aPDT: lasers (e.g., argon, diode, or neodymium-doped yttrium aluminum garnet [Nd: YAG] lasers), light-emitting diodes (LEDs), and incandescent lamps (e.g., quartz-tungsten-halogen or xenon-discharge lamps) [52,59]. Each of the three light sources has its advantages and disadvantages. The laser has the advantages of monochromaticity, high efficiency, and spectral stability; it can accurately match the peak absorption of different PSs, reduce energy loss and heat transfer to healthy tissues, efficiently couple into a single fiber, and can be installed on other light transmission devices. Laser systems are generally more expensive than LEDs. The halogen lamp has the advantage of spectral filtration to match any PSs, and the disadvantage is that it cannot effectively couple into the fiber bundle or liquid light guide. High heat causes significant light energy loss and quickly leads to high-temperature damage to healthy tissues. LEDs offer the advantages of being small, having a long service life, being cost-effective, providing scalable output power, and being easy to configure across different irradiation geometries. However, LEDs exhibit larger beam divergence and a broader emission spectrum than lasers. While a broader spectrum can be advantageous if it covers a wide PS absorption band, energy residing outside the target absorption profile yields negligible photochemical activity and may cause unwanted energy loss and localized thermal strain [33]. So it is also essential to make improvements to light sources.

#### 3.2.1. Visible and Water-Filtered Infrared Radiation

**Figure 4:**
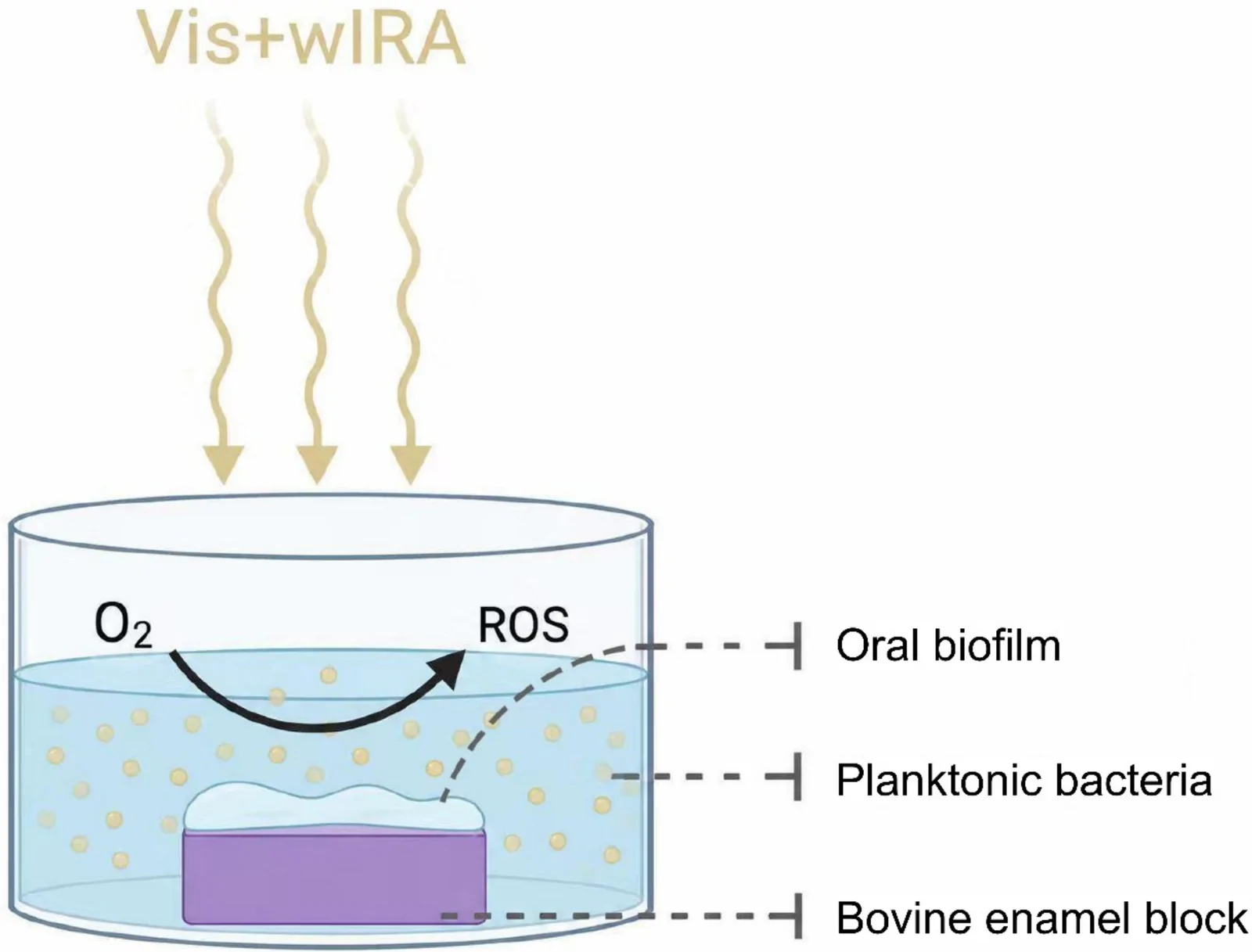
Schematic representation of the in vitro oral biofilm model treated with VIS + wIRA-mediated aPDT. ROS generated by light activation target both planktonic bacteria and oral biofilms cultured on bovine enamel blocks.

In dental aPDT, thermal safety is a critical parameter due to the vulnerability of oral soft and hard tissues. The average oral temperature in healthy adult humans is 35.73 to 37.41 °C [60]. After 45–60 s irradiation with a conventional halogen lamp, dentin temperature increases by 5 °C [61], which may cause damage to the pulp. In contrast, with irradiation using a combination of visible light (VIS) and water-filtered infrared radiation (wIRA) for up to 20 min, the tissue temperature increases by less than 3 °C [61]. VIS + wIRA also shows advantages over conventional aPDT light sources in terms of bactericidal activity (Figure 4). aPDT with VIS + wIRA is mainly used for oral-associated bacteria (Supplementary Table S1) [62,63,64,65,66,67,68,69].

#### 3.2.2. Laser and LED Co-Irradiation

Most existing studies on aPDT have used a single PS with light sources corresponding to specific wavelengths. Still, Pourhajibagher et al. [70] proposed that combining two photosensitizers with different light sources may enhance antimicrobial efficacy. They investigated a multifunctional system consisting of nano-curcumin and indocyanine green-metformin, activated by 450 nm laser irradiation and 810 nm LED irradiation, respectively, against *Enterococcus faecalis (E. faecalis)* biofilms. The dual-PS and dual-light strategy exhibited enhanced bactericidal activity compared with single-PS/single-light treatment, suggesting that combining different PS–wavelength pairs may provide a potential approach to improve aPDT performance.

However, the clinical translation of laser–LED co-irradiation remains limited. The relative contribution of each photosensitizer and wavelength is difficult to determine because appropriate dose-matched controls are often lacking. In addition, the increased system complexity, need for precise irradiation parameter optimization, and higher equipment requirements may restrict its application compared with simpler LED-based aPDT platforms. Further studies are needed to determine whether dual-light approaches provide clinically meaningful advantages over conventional single-wavelength irradiation.

Regarding clinical translation, dual-wavelength irradiation is not confined to laboratory settings. A CE-marked device combining 405 nm antibacterial blue light with 810 nm near-infrared aPDT, using indocyanine green as the photosensitizer, has already been evaluated in several randomized clinical trials for periodontitis, including in smokers, and for oral lichen planus. In these clinical studies, the rationale for combining wavelengths is not primarily to compensate for depth-dependent photon attenuation, but to superimpose two mechanistically distinct antibacterial actions, an ICG-independent bactericidal effect of blue light on endogenous bacterial chromophores, and ICG-mediated aPDT at 810 nm, both of which are most active near the biofilm surface, where clinically relevant oral pathogens predominantly reside; combining them has been reported to prevent the biofilm adaptation observed after repeated single-wavelength exposure. This clarifies, but does not contradict, the reviewer’s point: as noted above, the added value of the shorter-wavelength component remains concentrated at shallow depths, and at greater tissue depth the effect converges toward 810 nm aPDT alone. Dual-wavelength strategies are therefore best regarded as suited to superficial, biofilm-associated indications rather than as a general solution for deeper or thicker infected tissue.

Notably, a related but distinct dual-wavelength strategy, using a single photosensitizer rather than two—has already moved beyond in vitro study: a CE-marked device combining 405 nm antibacterial blue light with 810 nm aPDT has been evaluated in randomized clinical trials for periodontitis [71,72,73], where it reduced the biofilm adaptation seen after repeated single-wavelength exposure [74]. Because the two wavelengths differ markedly in tissue penetration, this added benefit is concentrated near the biofilm surface; at depth, the effect converges toward 810 nm aPDT alone. Dual-wavelength strategies are therefore best suited to superficial, biofilm-associated indications rather than a general depth-uniform solution.

#### 3.2.3. UV and Green Light

A more effective bactericidal effect can be achieved using two light sources in aPDT. Pujari AK et al. coupled metalloporphyrins with lignin-based ZnOAL nanocomposites (ZnOAL and ZnOKL) to form hydrophilic nano-couples (ZnOAL@EP and ZnOKL@EP) and evaluated their bacteriostatic activity assisted by dual light (UV + green light) on *Escherichia coli*. The photoinactivation potential displayed the highest fluorescence intensity when exposed to dual light (UV and green light) and the highest bactericidal activity due to higher ROS generation capacity [75]. So, using two or more PSs and light sources in a PDT is a research direction and may effectively compensate for the deficiencies of different PSs and light sources.

Advanced light-source strategies also carry practical limitations. UCNP-mediated upconversion typically suffers from low energy-conversion efficiency, meaning only a small fraction of incident NIR photons are converted into usable UV/visible output, which may limit in vivo ROS yield despite promising in vitro results. DL-aPDT offers the advantage of low equipment requirements; however, its clinical applicability remains limited by several practical constraints. Ambient sunlight varies substantially with weather conditions, latitude, season, and time of day, making it difficult to achieve consistent light fluence and reproducible treatment outcomes. In addition, the solar spectrum is not optimized for specific photosensitizers, and limited tissue penetration as well as prolonged exposure requirements may restrict its effectiveness for deeper or poorly accessible infections. These factors complicate dose standardization and may hinder the integration of DL-aPDT into routine clinical workflows.

## 4. Hypoxia Modulation

Conventional aPDT exerts its antibacterial effects through ROS generation, so adequate oxygen is generally required. However, subgingival and other deep-tissue infections are in an anoxic environment, and the effect of aPDT is inhibited. Therefore, improving an anoxic climate has become an important research direction to enhance the impact of aPDT [76,77].

### 4.1. H_2_O_2_

**Figure 5:**
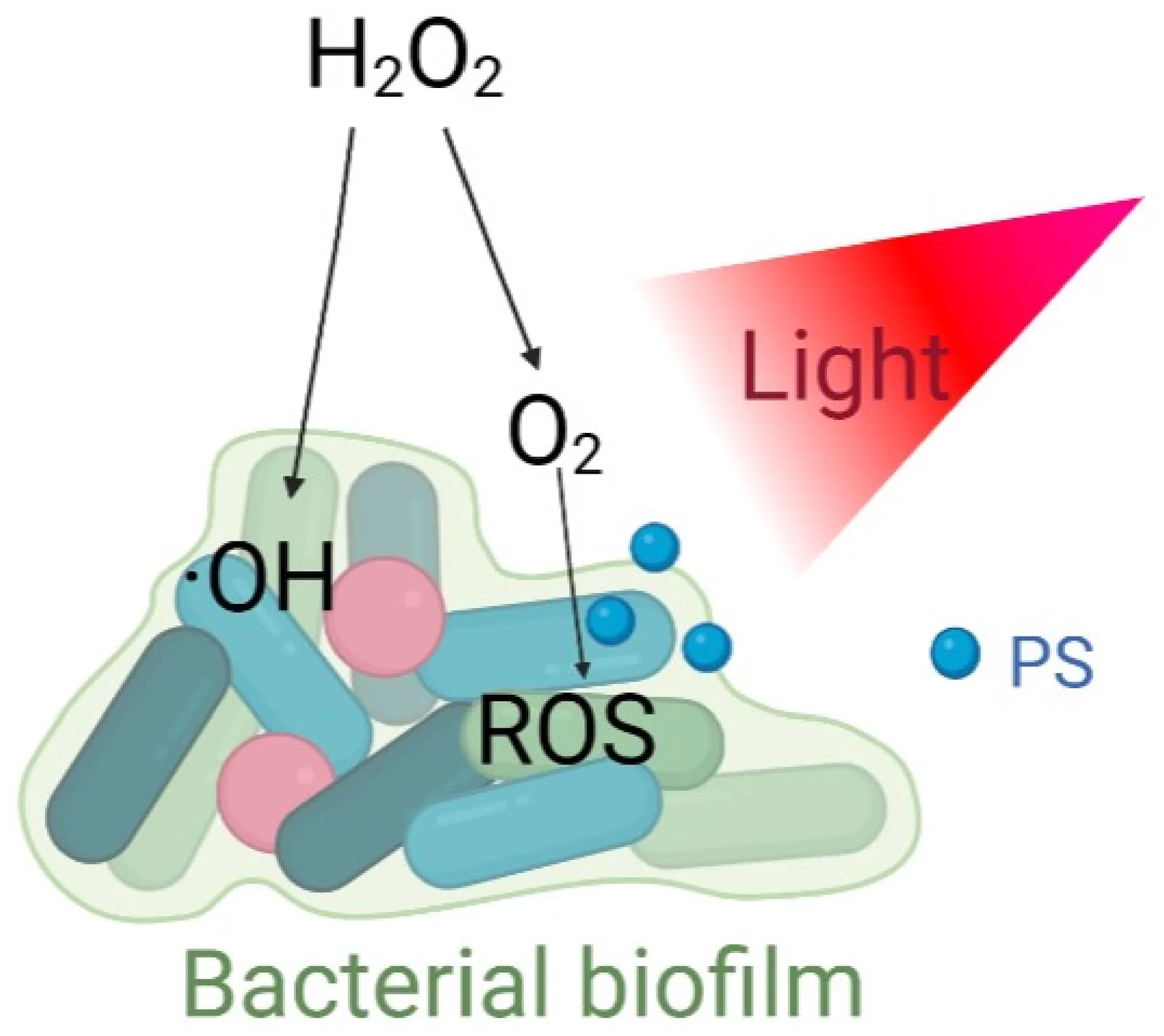
The function of H_2_O_2_ in aPDT. H_2_O_2_ contributes to aPDT through two mechanisms: it can decompose to release molecular oxygen, thereby alleviating hypoxia and promoting ROS generation by the photosensitizer, and it can be directly photolyzed into ·OH upon irradiation at specific wavelengths even in the absence of a photosensitizer.

H_2_O_2_ is a safe, strong oxidant that produces ·OH at specific wavelengths of light, destroying bacterial biofilms and allowing better penetration of PS [78,79]. In addition, under catalase-mediated decomposition (2H_2_O_2_ → 2H_2_O + O_2_), H_2_O_2_ can serve as a supplementary local oxygen source, potentially alleviating hypoxia and supporting ROS generation during aPDT (Figure 5); however, the magnitude of this oxygen contribution relative to tissue oxygen demand has not been directly quantified in the studies below and should be regarded as a proposed contributing mechanism rather than an established quantitative effect. Shirato M et al. added H_2_O_2_ photolysis to MB or TB-mediated aPDT, effectively increasing the antibacterial effects in *S. mutans* [80]. Garcez et al. compared the bactericidal efficacy of irradiation with MB in an aqueous solution and MB in an H_2_O_2_ solution in endodontic treatment [81]. They found that pretreatment with H_2_O_2_ effectively increased oxygen concentration while allowing PS to penetrate the bacterial membrane more effectively to kill *E. faecalis* and *Pseudomonas aeruginosa* and facilitate endodontic disinfection. Nie et al. [82] investigated that the combination of Ce6-aPDT with H_2_O_2_ has a much more significant bactericidal effect than Ce6-aPDT alone in oral microcosm biofilms, which is related not only to H_2_O_2_ itself but also to the large amount of oxygen produced by H_2_O_2_. Viana de Sousa et al. found that the application of H_2_O_2_ before aPDT affects the extracellular matrix (ECM) components, significantly reducing the polysaccharides (water-soluble polysaccharides, alkali-soluble polysaccharides), eDNA, and matrix proteins for *C. albicans* [83]. Because ECM can maintain the structural integrity of biofilms, protect microbial cells, and hinder the effects of antibacterial drugs and the penetration of PS, the efficiency of aPDT can be effectively improved after ECM is destroyed with H_2_O_2_.

Clinical translation has been reported for a related but mechanistically distinct approach. In a randomized controlled trial by Shirato et al., hydrogen peroxide photolysis, administered without an exogenous photosensitizer, was evaluated as an adjunct to root debridement in 53 patients with moderate-to-severe periodontitis [84]. The results demonstrated significant improvements in probing depth and bleeding on probing. However, that study employed H_2_O_2_ photolysis as a standalone antimicrobial therapy rather than as an oxygen-supplementation strategy combined with a separate photosensitizer. To the best of our knowledge, the specific photosensitizer-plus-H_2_O_2_ oxygen-boosting protocol described in the present work has not yet been assessed in any clinical photodynamic therapy trial.

### 4.2. Hypoxia-Tolerant aPDT: Emerging Type I and Oxygen-Independent-like Strategies

Adequate oxygen availability is traditionally considered essential for aPDT because most clinically used photosensitizers generate cytotoxic singlet oxygen (^1^O_2_) through a Type II photochemical pathway. Therefore, claims of “oxygen-independent” PDT should be interpreted cautiously. In most cases, these reports do not indicate that photodynamic reactions occur completely without oxygen, but rather that alternative oxygen-tolerant pathways may partially compensate for oxygen depletion.

The proposed Type III photochemical pathway represents one such hypothesis. Hamblin and Abrahamse suggested that certain PSs may maintain antimicrobial activity under anaerobic conditions based on observations from psoralen- and tetracycline-mediated PDT systems [85]. Subsequent studies have reported that several Type I-dominant PSs retain antibacterial activity under hypoxic conditions through electron-transfer reactions, generating oxygen-independent or less oxygen-dependent reactive species, such as superoxide radicals and hydroxyl radicals [86,87,88,89,90]. More recently, various hypoxia-tolerant strategies have been developed, including Type I-dominant photosensitizers, oxygen self-supplying systems, and ROS-regulating nanoplatforms, which aim to maintain photodynamic efficacy under oxygen-limited conditions [86,87,88,89,90,91,92].

Importantly, the reliability of oxygen-independent PDT studies should be evaluated in the context of experimental design. Many investigations demonstrating hypoxia-tolerant photodynamic activity have been performed using simplified in vitro models with isolated bacterial strains and chemically controlled anaerobic conditions. These systems are valuable for elucidating mechanisms but may not accurately reproduce the complex oxygen gradients, antioxidant defenses, extracellular polymeric substances, and microbial diversity present in clinical biofilms. Therefore, although hypoxia-tolerant PDT represents a promising strategy for overcoming oxygen limitation, further validation in physiologically relevant biofilm models and in vivo infection models is required before considering oxygen-independent PDT as a clinically established approach.

## 5. Adjunctive Strategies

Beyond optimizing the PS, light source, and oxygen supply, adjunctive strategies that chemically or physically augment the photodynamic reaction have attracted growing interest. These approaches do not replace the core aPDT triad but rather expand its therapeutic window by generating secondary toxic species, improving tissue penetration, or enhancing PS delivery.

### 5.1. Chemical Amplification of Photodynamic Reactions

Chemical amplification strategies aim to augment the quantum yield or cytotoxic potency of photogenerated ROS without altering the core PS–light–oxygen triad. Two principal approaches have emerged: (i) co-delivery of gaseous signaling molecules that participate in secondary redox chemistry, and (ii) addition of inorganic salts that convert primary ROS into more bactericidal secondary species. The following sections discuss representative examples of each strategy, focusing on their mechanisms, preclinical evidence, and translational feasibility.

#### 5.1.1. Use of Nitric Oxide in aPDT

Beyond its direct antibacterial effects, NO plays a mechanistically distinct and increasingly recognized role in potentiating aPDT by forming reactive nitrogen species (RNOS), particularly peroxynitrite (ONOO^−^). ONOO^−^ is generated by the diffusion-limited recombination of NO with superoxide anion (O_2_•^−^), a Type I photoproduct already present during aPDT, and exhibits substantially greater antibacterial potency than either precursor radical alone, causing lipid peroxidation, protein tyrosine nitration, and nucleic acid damage [93]. This coupling, therefore, represents a genuine synergistic mechanism rather than a simple additive effect: aPDT supplies the superoxide flux via Type I photochemistry, while co-delivered NO supplies the second radical required for ONOO^−^ formation, together yielding a more cytotoxic reactive species pool than either NO or aPDT alone could generate.

In a recent animal model of periodontal biofilm infection, co-delivery of nitric oxide (NO) was shown to enhance the antibacterial efficacy of aPDT [94], suggesting a potential supportive role for NO in antimicrobial photodynamic action; however, this finding is based on a single in vivo model and warrants further validation across other infection types. Nevertheless, NO’s poor stability limits its potential therapeutic applications. Yuan et al. found that combining NO with nanomaterials may solve this problem [95]. They presented an all-in-one phototherapeutic nanoplatform consisting of L-arginine, ICG, and mesoporous polydopamine. In this nanoplatform, L-arginine produces NO under NIR exposure, effectively destroys bacterial membranes, enhances the efficacy of aPDT, and facilitates rapid recovery of infected wounds. Hu et al. developed a nanocapsule linking NO- and Ce6-precursor drugs via α-cyclodextrin that releases NO to directly kill bacteria and reacts with photogenerated ROS to form ONOO^−^, while also depleting biofilm glutathione, thereby amplifying aPDT efficacy with minimal host toxicity [96]. Similarly, Wang et al. synthesized a self-assembled porphyrin nanoparticle in which NO is coordinated to the central zinc metal, releasing ONOO^−^ upon light irradiation to enhance bactericidal activity [97]. Collectively, these studies indicate that ONOO^−^ formation, rather than NO release per se, is the key synergistic mechanism underlying NO-potentiated aPDT, and future nanoplatform design should explicitly optimize the spatiotemporal co-localization of NO release and Type I ROS generation to maximize ONOO^−^ yield.

#### 5.1.2. Use of Inorganic Salt in aPDT

Chemical amplification strategies using inorganic salts have been explored to enhance aPDT efficacy by converting primary ROS into longer-lived secondary reactive species. Among these approaches, potassium iodide (KI) has attracted the greatest translational interest because of its established safety profile and ability to generate reactive iodine species that enhance bacterial killing [7,98]. Mechanistically, iodine species generated during KI-potentiated aPDT exhibit longer diffusion distances than conventional short-lived ROS, allowing enhanced antimicrobial effects beyond the immediate photosensitizer–light interaction zone.

Other inorganic salts, including potassium bromide, sodium thiocyanate, potassium selenocyanate, and sodium azide, have demonstrated ROS-modulating effects in vitro [7,10,99,100,101] (Table 1). However, these compounds remain primarily of mechanistic interest because of limited safety evaluation, insufficient in vivo validation, or potential toxicity concerns. Therefore, their contribution to clinical translation of aPDT remains uncertain.

Collectively, inorganic salt-based amplification represents a promising strategy for enhancing the antimicrobial efficacy of aPDT; however, future clinical development should prioritize agents with established safety profiles and realistic translational potential. For clinical implementation, efficacy enhancement must be balanced with biosafety, treatment reproducibility, and compatibility with existing clinical workflows.

### 5.2. Physical Approaches to Improve Treatment Accessibility

A recurrent limitation of conventional aPDT is the shallow penetration of visible and near-infrared light through biological tissues, which restricts effective treatment to superficial or optically accessible lesions. Physical adjunctive modalities that either bypass the optical penetration barrier or enhance PS delivery independent of light dosage, therefore, represent a distinct strategic axis. Two such approaches are discussed below: SDT, which substitutes acoustic for electromagnetic energy, and pulsed electric field (PEF), which facilitates PS permeation through membrane electroporation.

#### 5.2.1. Use of Sonodynamic Therapy in aPDT

**Figure 6:**
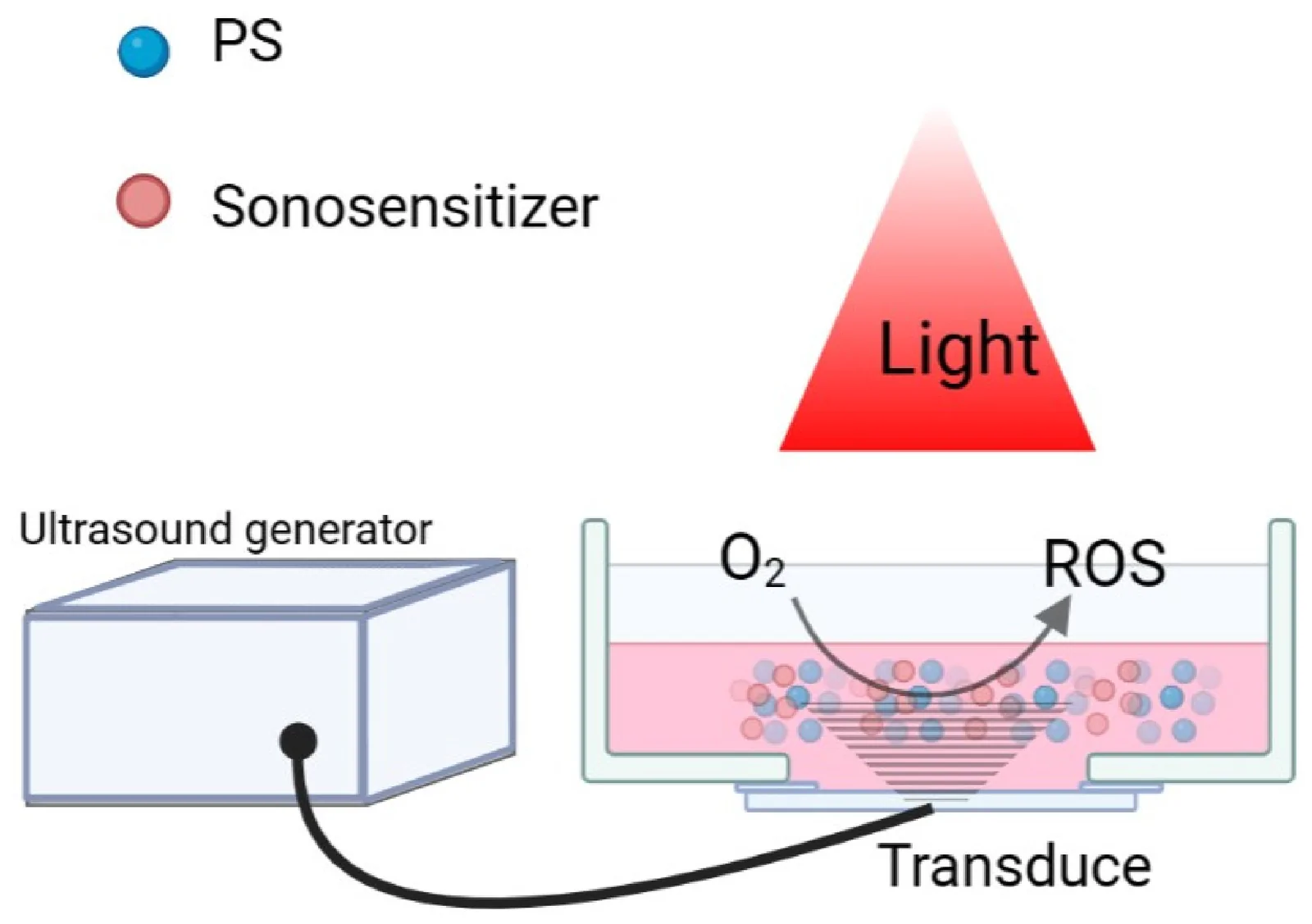
Schematic illustration of sonodynamic therapy device.

Sufficient depth of penetration into the tissue is essential for the clinical application of aPDT. SDT (Figure 6) with ultrasound has strong tissue penetration [102,103], which activates sonosensitizers that accumulate in the lesion, producing ROS that destroy the bacterial cell wall and membrane. Tetrapyrrole macrocycles and porphyrins are currently the most extensively investigated classes of sonosensitizers. It has been shown that SDT, in collaboration with aPDT, can inactivate *Staphylococcus aureus*, *P. aeruginosa* [104], *C. albicans* [105], and *E. faecalis* [106] more efficiently. Despite this preclinical promise, clinical translation of SDT-aPDT remains at an early stage (Table 2): clinically validated sonosensitizers are scarce, and ultrasound parameters effective against biofilms must be carefully optimized to avoid thermal tissue injury, as discussed further below.

#### 5.2.2. Use of Pulsed Electric Field in aPDT

**Figure 7:**
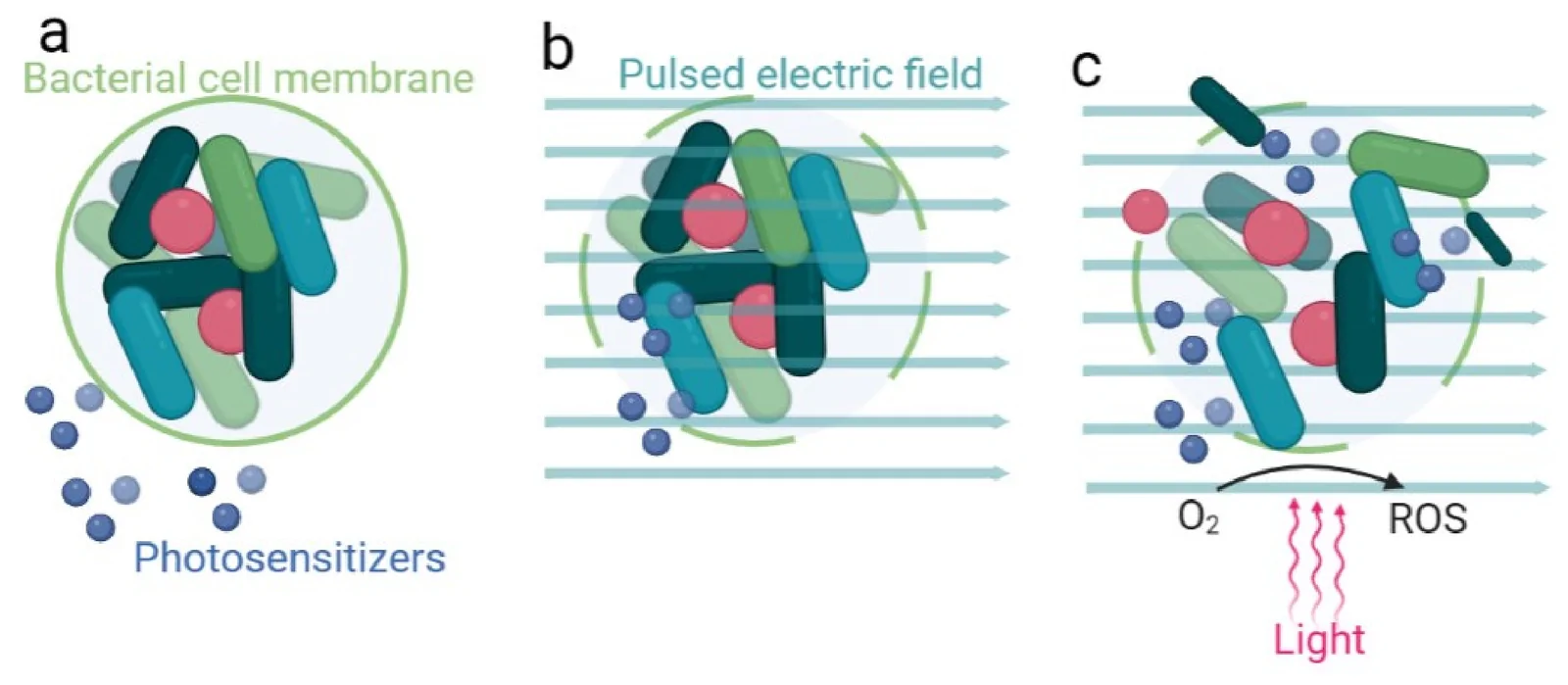
Hypothetical mechanism of action by the association of aPDT and pulsed electric field (PEF). (**a**) The bacterial cell membrane prevents the PS from penetrating. (**b**) Application of PEF permeabilizes PS diffusion through the bacterial cell membrane. (**c**) The application of visible light corresponds to the PS activation for ROS production, and the PEF promotes the production of ROS.

In a review of PEF-assisted aPDT, De Melo et al. summarized in vitro studies reporting that this combination markedly enhances bactericidal efficacy against biofilm-forming bacteria [52]. First, when cells are exposed to a high-power external PEF, cell membrane permeability to various impermeable molecules increases suddenly. This process is called electroporation or electropermeabilization, which can promote PS infiltration. The underlying mechanism involves the induction of transient membrane electroporation by the external electric field. Specifically, PEF generates a transmembrane potential that promotes dipole polarization and the reorientation of polar molecules within the membrane. When the electric field exceeds a critical threshold, transient nanopores are formed, increasing bacterial membrane permeability and facilitating PS penetration. Meanwhile, membrane disruption caused by PEF may further enhance ROS-mediated bacterial damage (Figure 7).

However, clinical translation of PEF-assisted aPDT remains challenging. Current systems are primarily designed for experimental settings, and clinically applicable devices need further optimization to deliver electric fields locally, safely, and reproducibly while minimizing patient discomfort. Therefore, PEF-assisted aPDT may be more suitable for accessible infections, such as oral biofilms or superficial wounds, where localized application is feasible.

In summary, SDT and PEF address the accessibility bottleneck of aPDT using fundamentally different physical principles: acoustic energy propagation and electric-field–mediated membrane permeabilization, respectively. Preclinical data suggest that both modalities synergize with photodynamic therapy, yet their integration into clinical workflows poses distinct engineering challenges: SDT requires optimization of sonosensitizer pharmacokinetics and ultrasound parameter sets to avoid thermal tissue damage, whereas PEF requires precise control of field strength and pulse duration to achieve reversible electroporation without irreversible cell lysis. Importantly, neither modality eliminates the need for PS and oxygen; rather, they expand the spatial and kinetic dimensions within which the photodynamic reaction can be initiated. Hybrid devices capable of delivering light, ultrasound, and an electric field sequentially or simultaneously may ultimately offer the most versatile platform for treating deep-seated or refractory infections.

SDT and PEF face distinct scalability barriers. Clinically validated sonosensitizers remain scarce, and ultrasound parameters effective against biofilms may risk thermal injury to adjacent tissue if not carefully calibrated. PEF devices require precise, tissue-specific control of field strength and pulse duration; miniaturized, clinic-ready PEF applicators compatible with simultaneous light delivery have not yet been developed, and the pain or discomfort associated with electroporation in accessible tissues (e.g., oral mucosa) has not been systematically assessed.

## 6. Clinical Barriers and Translational Strategies

Although the preceding sections outline numerous strategies for enhancing the antimicrobial efficacy of aPDT, translating these advances from bench to bedside faces systematic barriers that are largely independent of any single strategy’s mechanism of action. This section examines these barriers along three dimensions: regulatory classification, manufacturing and protocol standardization, and clinical workflow integration, and evaluates which of the strategies discussed above are most likely to reach clinical implementation in the near term.

### 6.1. Regulatory Classification and Approval Pathway

Unlike conventional pharmaceuticals, aPDT constitutes a drug–device combination product, in which the PS and the light source are typically subject to independent, and sometimes divergent, regulatory review. In the United States, the PS is evaluated by the Center for Drug Evaluation and Research, while the corresponding light-delivery device falls under the Center for Devices and Radiological Health, requiring coordinated submissions through the FDA Office of Combination Products [107]. Comparable but non-identical frameworks exist in the European Union, where combination products are classified according to primary mode of action under the Medical Device Regulation and pharmaceutical legislation, creating discrepancies in premarket and post-marketing surveillance requirements between jurisdictions [108]. This regulatory duality disproportionately disadvantages the more structurally complex strategies discussed above: multifunctional nanoplatforms that combine a photosensitizer with stimuli-responsive carriers, imaging agents, or a second therapeutic modality may be classified as combination products with additional constituent parts, further complicating and prolonging regulatory review relative to a single-agent PS with a standard light source.

### 6.2. Manufacturing Scalability and Protocol Standardization

A second barrier concerns the reproducibility of both the therapeutic agent and the treatment protocol. Nanomaterial-based PS carriers, stimuli-responsive platforms, and multifunctional nanoplatforms generally require multi-step synthesis with batch-to-batch variability in particle size, surface functionalization, and drug loading, properties that must remain within tight tolerances for consistent photodynamic performance but that have rarely been assessed under Good Manufacturing Practice conditions in the studies reviewed here. Compounding this, aPDT protocols across the reviewed literature vary substantially in PS concentration, drug–light interval, irradiance, total light dose, and light source type, making cross-study comparison of efficacy difficult and complicating the design of standardized clinical trial protocols. By contrast, strategies with simpler, single-component formulations and well-established dosimetry, such as potassium iodide potentiation or conventional MB/toluidine blue-mediated aPDT, face substantially fewer standardization hurdles and are correspondingly closer to routine clinical protocols.

### 6.3. Clinical Workflow Integration and Cost-Effectiveness

A third barrier is practical integration into existing clinical workflows. Multimodal physical approaches such as sonodynamic therapy and pulsed electric field co-treatment require dedicated equipment, operator training, and additional treatment time beyond conventional light-based aPDT, which may limit their adoption in general dental or wound-care settings where cost-effectiveness concerns have already been raised regarding adjunctive PDT [5,6]. Similarly, upconversion nanoparticle- and dual-wavelength-based light source strategies depend on specialized light sources that are considerably more expensive than standard LED units, restricting their near-term accessibility to specialized centers rather than primary care settings.

### 6.4. Which Strategies Are Best Positioned for Near-Term Clinical Translation

Considered together, strategies that (i) rely on single, well-characterized components, (ii) use existing regulatory precedent, and (iii) require no specialized equipment beyond conventional light sources appear best positioned for near-term clinical adoption. These include inorganic salt potentiation with potassium iodide, given its long history of human use and compatibility with already-approved PS [98,109,110,111], and daylight-mediated aPDT, which has already achieved clinical adoption in dermatology and requires no novel device approval [57,58]. Conversely, multifunctional nanoplatforms, stimuli-responsive delivery systems, and combined physical-modality approaches, while mechanistically promising and supported by encouraging preclinical data, are likely to require considerably longer development timelines due to the compounded regulatory, manufacturing, and cost barriers outlined above. Bridging this gap will require early engagement with regulatory bodies regarding combination product classification, adoption of standardized reporting of PS dosimetry and light parameters across preclinical studies, and structured cost-effectiveness analyses comparing aPDT-based regimens with existing antimicrobial standards of care.

## 7. Conclusions and Prospects

Over the past decade, aPDT has evolved from a conventional PS-light-oxygen system into a multifunctional antimicrobial platform. As summarized in this review, advances in photosensitizer design, nanomaterial- and non-nanomaterial-based delivery systems, optimized light sources, oxygen-regulating strategies, and synergistic therapeutic approaches have collectively enhanced bacterial targeting, biofilm penetration, ROS generation, and antimicrobial efficacy. These advances have expanded the therapeutic potential of aPDT; however, they should be regarded as approaches to optimize treatment performance rather than complete solutions to the intrinsic limitations of photodynamic therapy.

This emerging paradigm provides new opportunities for the clinical translation of aPDT, while also highlighting several key considerations that will influence its future clinical adoption. Based on the current evidence, three unresolved bottlenecks deserve particular attention. First, biocompatibility remains the primary concern for advanced antimicrobial platforms. Although multifunctional nanomaterials and stimuli-responsive systems have significantly enhanced antibacterial efficacy in preclinical studies, their long-term biosafety, biodegradation pathways, and potential systemic toxicity remain poorly understood [112]. The increasing structural complexity of multifunctional platforms may further complicate large-scale manufacturing and regulatory approval, underscoring the need to balance therapeutic performance with material safety [113].

Second, limited treatment depth is a major obstacle. The effectiveness of aPDT is intrinsically constrained by the penetration depth of excitation light and the restricted diffusion distance of ROS. In addition, the hypoxic microenvironment and dense extracellular polymeric matrix of mature biofilms further reduce ROS generation and hinder photosensitizer delivery, making deep-seated or highly structured infections difficult to eradicate completely [114]. Although oxygen-generating systems and near-infrared-responsive platforms have shown encouraging results, their clinical effectiveness requires further validation [115].

Finally, the lack of standardized treatment protocols continues to impede clinical adoption. Considerable variations in photosensitizer selection, light source parameters, irradiation dose, treatment duration, and outcome evaluation make it difficult to compare results across studies or establish evidence-based clinical guidelines [116]. Future clinical translation will therefore require not only technological innovation but also standardized protocols, high-quality clinical trials, and consensus recommendations to ensure reproducible and predictable therapeutic outcomes.

Addressing these three bottlenecks must become the primary focus of next-generation aPDT research. From our perspective, four specific strategies represent the most promising opportunities to overcome these translational barriers while maintaining strong clinical relevance. Foremost among these are stimuli-responsive multifunctional platforms. These intelligent systems enable on-demand activation of PS in response to pathological conditions such as acidic pH, elevated ROS, enzyme overexpression, or hypoxia [117]. Compared with conventional delivery systems, they improve photosensitizer accumulation, biofilm penetration, and localized ROS generation while minimizing damage to surrounding healthy tissues. Integrating multiple therapeutic functions into a single platform provides a practical strategy for overcoming several biological barriers simultaneously, making this approach highly attractive for future precision antimicrobial therapy [118].

In tandem with responsive delivery, oxygen-regulating strategies are crucial to overcoming hypoxia, which remains one of the principal factors limiting ROS generation and antibacterial efficacy. Strategies that improve local oxygen availability, including oxygen-generating nanomaterials, catalase-mimicking nanozymes, and oxygen-carrying delivery systems, are expected to substantially improve therapeutic performance against mature biofilms and deep-seated infections. Recent studies indicate that overcoming oxygen deficiency may become a key prerequisite for extending aPDT beyond superficial infections and improving treatment consistency under complex pathological conditions [112].

Third, increasing evidence suggests that formulating multifunctional synergistic therapies can significantly enhance outcomes. Rather than relying solely on ROS generation, combining aPDT with complementary therapeutic modalities, including photothermal therapy, chemodynamic therapy, nitric oxide therapy, sonodynamic therapy, or antimicrobial agents, can achieve synergistic antibacterial activity while reducing the required light dose and photosensitizer concentration [119]. Such combination strategies improve biofilm disruption and broaden the therapeutic spectrum, making them particularly promising for managing multidrug-resistant and biofilm-associated infections.

However, while numerous advanced technologies have demonstrated encouraging antibacterial performance, future progress relies heavily on prioritizing clinically translatable and standardized therapeutic systems. Translating these innovations to the clinic will depend not only on developing complex multifunctional materials but also on improving reproducibility, scalable manufacturing, and regulatory compliance [120]. Clinically applicable systems based on established PS and optimized light-delivery protocols may provide the fastest route toward broader implementation. Taken together, these four strategies share a common objective: shifting the development of aPDT from merely maximizing antibacterial activity in experimental models toward achieving safe, reproducible, and translatable therapeutics.

Although aPDT has been investigated for decades and has demonstrated clinical feasibility in selected applications, its integration into routine healthcare practice remains limited. This gap does not primarily reflect the absence of technological innovation, but rather several practical barriers, including insufficient clinician awareness, limited incorporation of photodynamic therapy into medical and dental education, inconsistent treatment protocols, restricted reimbursement pathways, and insufficient high-quality clinical evidence supporting cost-effectiveness. Importantly, aPDT should not be considered a replacement for systemic antimicrobial therapy, as its therapeutic advantages are mainly applicable to localized, accessible, and biofilm-associated infections. Therefore, future efforts should focus on defining appropriate clinical indications, improving professional training, establishing standardized treatment protocols, and generating robust clinical evidence to identify scenarios in which aPDT represents a method of choice rather than merely an adjunctive approach.

## Figures and Tables

**Table 1:** Inorganic Salt Potentiation of aPDT.

Salt	Proposed Mechanism	Compatible PSs	Main Effect	Clinical Feasibility
Potassium iodide (KI)	^1^O_2_/·OH oxidizes I^−^ to reactive iodine species (I_3_^−^, I_2_, HOO·)	MB [92], TiO_2_ [89], rose bengal [93], porphyrin [94], eosin [93], TBO [26]	Substantially enhanced bactericidal effect across multiple PS classes	High—established clinical safety history at topical concentrations [89]
Potassium bromide (KBr)	Oxidation of Br^−^ to hypobromite (Type I pathway)	TiO_2_ [87,88,89,90,91]	Enhanced bactericidal effect, weaker than KI (Br^−^ harder to oxidize)	Low—limited evidence base
Sodium azide (NaN_3_)	Oxidation of N_3_^−^ to azide radicals (N_3_·) despite azide’s known role as a ^1^O_2_ quencher	Phenothiazinium dyes [89]	Paradoxical enhancement of bactericidal effect	Not viable—systemic mammalian toxicity; mechanistic interest only [89]
Sodium thiocyanate (KSCN)	SCN^−^ oxidized via H_2_O_2_ (two-electron) or directly by ^1^O_2_	Phenothiazinium salts only [87,88,89,90,91]	Bactericidal via sulfite/sulfur trioxide radical intermediates	Low—narrow PS compatibility
Potassium selenocyanate (KSeCN)	SeCN^−^ oxidized by ^1^O_2_ to selenocyanogen	Broader range: porphyrin, MB, rose bengal [87,88,89,90,91]	More potent than KSCN; broader applicability	Low—limited safety data

**Table 2:** Risk–Benefit Assessment of Strategies to Enhance aPDT Efficacy.

**Tier 1—Near-term clinical feasibility**					
Strategy	Primary Reported Benefit	Key Limitations/Risks	Scalability and Cost	Level of Evidence	Translational Readiness
DL-aPDT	Low cost; accessible outside clinical settings	Dependent on weather/season; dosimetry difficult to standardize	High (cost)/Low (reproducibility)	Clinical (dermatology)	Moderate (indication-specific)
Inorganic salt potentiation (KI)	Enhances bactericidal ROS; established human safety profile	Efficacy/dosing window not standardized across PS types	High—low cost, established clinical history	In vitro + limited human	Moderate
**Tier 2—Moderate-term, in vivo validated**					
Strategy	Primary Reported Benefit	Key Limitations/Risks	Scalability and Cost	Level of Evidence	Translational Readiness
Liposomal/micellar PS carriers	Improved solubility, reduced aggregation, higher bioavailability	Limited long-term stability data; potential immune clearance in vivo	Moderate—established pharmaceutical technology	In vitro + limited in vivo	Moderate
Laser/dual-wavelength light sources	Precise wavelength matching; higher photochemical efficiency	Substantially higher equipment cost than LED; limited access in low-resource settings	Low (cost)	In vitro + limited clinical	Moderate
H_2_O_2_-assisted aPDT	Alleviates local hypoxia; enhances biofilm/ECM disruption	Cytotoxic to host tissue at bactericidal concentrations if not precisely dosed	High—inexpensive, widely available	In vitro + limited in vivo	Moderate
Antibiotic–PS conjugates	High target selectivity; claimed resistance-independent killing	Sub-lethal antibiotic exposure may still contribute to selection pressure (not directly tested)	Moderate	In vitro	Low–Moderate
**Tier 3—Early stage/mechanistic value only**					
Strategy	Primary Reported Benefit	Key Limitations/Risks	Scalability and Cost	Level of Evidence	Translational Readiness
Antibody/peptide-conjugated (3rd-gen) PS	High bacterial selectivity; reduced off-target phototoxicity	Complex multi-step conjugation chemistry; batch variability; immunogenicity unassessed	Low—GMP manufacturing not demonstrated	In vitro only	Low
Metal oxide/UCNPs	Deep-tissue ROS generation via NIR-to-UV/Vis conversion	Heavy-metal content; uncharacterized biodegradation; low conversion efficiency	Low—multi-step synthesis, high cost	*In vitroIn vitro* + small-animal	Low
Stimuli-responsive nanoplatforms	Site-specific PS release; reduced off-target ROS	High structural complexity; in vitro conditions may not replicate *in vivoin vivo* microenvironment	Low	In vitro predominant	Low
Exosome-based PS delivery	Inherent biocompatibility; intrinsic antimicrobial peptide content	No standardized isolation protocol; yield/reproducibility unestablished	Low	Early in vitro	Low
Efflux pump inhibitors	Increased intracellular PS accumulation	Host–cell toxicity at effective concentrations not systematically characterized	Moderate	In vitro	Low
Hypoxia-tolerant PS (Se-/charge-engineered)	Maintains ROS generation under anaerobic conditions	Multi-step synthesis; dark toxicity/photostability assessed only in single models	Low	In vitro + single-model in vivo	Low
NO co-delivery	Enhances membrane disruption and biofilm penetration	NO instability requires nanoplatform stabilization; single in vivo model only	Low	Single in vivo model	Low
SDT synergy	Deep tissue penetration via acoustic energy	Risk of thermal tissue damage; limited clinically validated sonosensitizers	Low–Moderate	In vitro predominant	Low
PEF synergy	Enhances PS membrane permeation via electroporation	Device miniaturization/clinical integration immature; patient tolerability unassessed	Low	In vitro/ex vivo	Low
Inorganic salt potentiation (NaN_3_ and others) ^a^	Illustrates mechanistic ROS-potentiation principles	Systemic toxicity precludes clinical use	Not applicable	In vitro only	Not translatable

^a^ Included as a mechanistic comparator only, not as a candidate clinical strategy. Abbreviation: UCNPs, upconversion nanoparticles; DL-aPDT, Daylight-based aPDT; SDT, Sonodynamic therapy; PEF, Pulsed electric field.

## Data Availability

Data sharing is not applicable to this article as no new data were created or analyzed in this study.

## Supplementary Materials

The following supporting information can be downloaded at: https://www.mdpi.com/article/10.3390/antibiotics15080748/s1, Table S1. Updates on VIS+wIRA-mediated aPDT for the oral microbiome.
