# Supplementary material for: Strategies to Enhance the Efficacy and Clinical Translation of Antimicrobial Photodynamic Therapy
Source: Antibiotics (Basel). 2026 Aug 2;15(8):748. doi: 10.3390/antibiotics15080748 (PMC13510149; doi:10.3390/antibiotics15080748)
Supplement: Supplementary file 1 [file antibiotics-15-00748-s001.zip › antibiotics-4429541-supplementary.pdf]

Supplementary Table S1. Updates on VIS+wIRA-mediated aPDT for the oral microbiome.

Ref.	PS	Concentration (μg/mL)	Irradiation time (min)	Irradiance of VIS (mW/cm²)	Irradiance of wIRA (mW/cm²)	Bacterium	Bacterial morphology	Incubation time	Killing rate	Average viability of negative control group	aPDT (VISwIRA) mean viability
Al-Ahmad A[1-3]	Ce6	100	5	48	152	Eight periodontitis-associated microorganisms cultured in the laboratory;	Planktonic monobiont	0	> 99.9%	/	/
						subgingival plaque in chronic periodontitis and	Biofilm	0	99.9% Aerobes, 99.9% Anaerobes	37.2%	2.9%
						supragingival plaque in healthy volunteers	Biofilm	2 h<br>3 d	> 99.9%<br>98.1%	/<br>/	/<br>/
	TB	100	5	48	152	Supragingival plaque in	Biofilm	2 h<br>3 d	> 99.9%<br>99.4%	/	/

						healthy volunteers					
		5-50	1	48	152	Saliva of healthy volunteers	Saliva	0	> 99.9%		
						Laboratory cultured Enterococcus faecalis (E. faecalis) and Streptococcus mutans (S. mutans)	Planktonic monobiont	0	99.0% E. faecalis<br>98.0% S. mutans		
Karygianni L[4, 5]	Tetrahydroporphyrin-tetratosylate	100	5	48	152	Supragingival plaque in healthy volunteers	Biofilm	2 h<br>3 d	> 99.9%<br>> 99.9%	71.2%<br>81.9%	9.4%<br>25.7%
	Ce6	100	5	48	152	Supragingival plaque in healthy volunteers	Biofilm	2 h<br>3 d	> 99.9%<br>99.9%	74.5%<br>82.1%	12.1%<br>10.4%
	TB	100	5	48	152	Supragingival plaque in healthy	Biofilm	2 h<br>3 d	> 99.9%<br>99.9%	74.5%<br>82.1%	20.7%<br>17.7%

						volunteers					
Vollmer A[6]	H. perforatum extract	32000	5	48	152	Supragingival plaque in healthy volunteers	Biofilm	2 h<br>3 d	100%	/	/
Burchard T[7]	ICG	50-500	5	48	152	Supragingival plaque in healthy volunteers;	Biofilm	2 h	100% (450 μg/mL)	/	/
								3 d	> 99.9% (450 μg/mL)		
						saliva of healthy volunteers	Saliva	0	> 99.9% (300 μg/mL)		
Solarte DLG[8]	ICG	50-500	5	48	152	Eight periodontitis-associated microorganisms cultured in the laboratory;	Planktonic monobiont	0	> 99.0% (all concentrations)	/	/
						subgingival plaque in chronic periodontitis	Biofilm	0	> 90.0% (50 μg/mL); < 90.0% (150 μg/mL, 500 μg/mL)	/	/

1. Al-Ahmad A, Walankiewicz A, Hellwig E, et al. Photoinactivation Using Visible Light Plus Water-Filtered Infrared-A (vis+wIRA) and Chlorine e6 (Ce6) Eradicates Planktonic Periodontal Pathogens and Subgingival Biofilms. *Front Microbiol.* 2016;7:1900.
2. Al-Ahmad A, Bucher M, Anderson AC, et al. Antimicrobial Photoinactivation Using Visible Light Plus Water-Filtered Infrared-A (VIS + wIRA) Alters In Situ Oral Biofilms. *PLOS ONE.* 2015;10(7):e0132107.
3. Al-Ahmad A, Tennert C, Karygianni L, Wrbas K, Hellwig E, Altenburger M. Antimicrobial photodynamic therapy using visible light plus water-filtered infrared-A (wIRA). *Journal of medical microbiology.* 2013;62(3):467-473.
4. Karygianni L, Ruf S, Hellwig E, Follo M, Vach K, Al-Ahmad A. Antimicrobial Photoinactivation of In Situ Oral Biofilms by Visible Light Plus Water-Filtered Infrared A and Tetrahydroporphyrin-tetratosylate (THPTS). *Microorganisms.* Jan 11 2021;9(1).
5. Karygianni L, Ruf S, Hellwig E, Follo M, Vach K, Al-Ahmad A. Antimicrobial Photoinactivation of In Situ Oral Biofilms by Visible Light Plus Water-Filtered Infrared A and Tetrahydroporphyrin-tetratosylate (THPTS). *Microorganisms.* 2021;9(1):145.
6. Vollmer A, Al-Ahmad A, Argyropoulou A, et al. Antimicrobial Photoinactivation Using Visible Light Plus Water-Filtered Infrared-A (VIS + wIRA) and Hypericum Perforatum Modifies In Situ Oral Biofilms. *Sci Rep.* Dec 30 2019;9(1):20325.
7. Burchard T, Karygianni L, Hellwig E, Wittmer A, Al-Ahmad A. Microbial Composition of Oral Biofilms after Visible Light and Water-Filtered Infrared a Radiation (VIS+wIRA) in Combination with Indocyanine Green (ICG) as Photosensitizer. *Antibiotics (Basel).* Aug 23 2020;9(9).
8. Solarte DLG, Rau SJ, Hellwig E, Vach K, Al-Ahmad A. Antimicrobial Behavior and Cytotoxicity of Indocyanine Green in Combination with Visible Light and Water-Filtered Infrared A Radiation against Periodontal Bacteria and Subgingival Biofilm. *Biomedicines.* Apr 20 2022;10(5).
